# *KIT* Somatic Mutations and Immunohistochemical Expression in Canine Oral Melanoma

**DOI:** 10.3390/ani10122370

**Published:** 2020-12-10

**Authors:** Ginevra Brocca, Beatrice Poncina, Alessandro Sammarco, Laura Cavicchioli, Massimo Castagnaro

**Affiliations:** 1 Department of Comparative Biomedicine and Food Science, University of Padua, Legnaro, 35020 Padua, Italy; beatriceponcina@gmail.com (B.P.); alessandro.sammarco@unipd.it (A.S.); laura.cavicchioli@unipd.it (L.C.); massimo.castagnaro@unipd.it (M.C.); 2 Department of Neurology and Radiology, Massachusetts General Hospital, Harvard Medical School, Boston, MA 02129, USA

**Keywords:** Canine oral melanoma (COM), copy number aberration (CNA), dog, immunohistochemistry (IHC), single nucleotide polymorphism (SNP), receptor tyrosine kinase (KIT)

## Abstract

**Simple Summary:**

Malignant melanomas arising from mucosal sites are very aggressive neoplastic entities which affect both humans and dogs. The family of tyrosine kinase receptors has been increasingly studied in humans for this type of neoplasm, especially the gene coding for the proto-oncogene *KIT*, and tyrosine kinase inhibitors are actually available as treatment. However, *KIT* alteration status in canine oral melanoma still lacks characterization. In this study, we investigated the mutational status and the tissue expression of *KIT* through DNA sequencing and immunohistochemical analysis, respectively. A homogeneous cohort of 14 canine oral melanomas has been collected, and while tissue expression of the protein was detected, no mutations were identifiable, most likely attributing the dysregulation of this oncogene to a more complex pattern of genomic aberration.

**Abstract:**

Canine oral melanoma (COM) is an aggressive neoplasm with a low response to therapies, sharing similarities with human mucosal melanomas. In the latter, significant alterations of the proto-oncogene *KIT* have been shown, while in COMs only its exon 11 has been adequately investigated. In this study, 14 formalin-fixed, paraffin-embedded COMs were selected considering the following inclusion criteria: unequivocal diagnosis, presence of healthy tissue, and a known amplification status of the gene *KIT* (seven samples affected and seven non-affected by amplification). The DNA was extracted and *KIT* target exons 13, 17, and 18 were amplified by PCR and sequenced. Immunohistochemistry (IHC) for KIT and Ki67 was performed, and a quantitative index was calculated for each protein. PCR amplification and sequencing was successful in 97.62% of cases, and no single nucleotide polymorphism (SNP) was detected in any of the exons examined, similarly to exon 11 in other studies. The immunolabeling of KIT was positive in 84.6% of the samples with a mean value of 3.1 cells in positive cases, yet there was no correlation with aberration status. Our findings confirm the hypothesis that SNPs are not a frequent event in *KIT* activation in COMs, with the pathway activation relying mainly on amplification.

## 1. Introduction

The proto-oncogene *KIT*, firstly identified as a homolog of the feline sarcoma viral oncogene *v-kit* [1], encodes for a type 3 receptor tyrosine kinase (KIT) and it is expressed in a wide range of healthy cells [2,3]. Even if its precise role is not completely understood [4], *KIT* is also normally expressed in the development of melanocytes [2] and it is involved in melanogenesis and melanocyte survival during migration from the neural crest to the cutis. It appears to be more involved in melanocyte migration rather than proliferation [5] with interesting implications since *KIT* mutant melanocytes could acquire elevated migration abilities. Indeed, given its key role, the gene coding for the KIT protein (*KIT*) received great attention in the field of human oncology, particularly in the study of neoplasms of melanocytic origin. The genomes of both human cutaneous and mucosal melanomas were deeply studied, and human mucosal melanomas (hMMs) showed a higher frequency of aberrations in the *KIT* gene rather than their cutaneous counterparts [3,6,7,8,9]. HMMs are affected by both structural alterations, such as copy number aberrations (CNAs), and single nucleotide polymorphisms (SNPs) of *KIT*, with CNAs in 26.3% of cases [6,8] and SNPs in 7–23% [4,6,7,8,9,10,11,12,13]. Therefore, *KIT* mutations have been proposed as an adverse prognostic factor [13]. On the contrary, in cutaneous melanomas, *KIT* is affected by CNAs in 6.7% of cases and by SNPs in 1.7% of cases [8].

Mutations identified in hMMs mainly affected exon 11 (Ex-11) [8,9], where four hotspots have been identified as driver mutations [7].

In humans, most tumors affected by *KIT* mutations [3,14] mainly show SNPs affecting Ex-11 (65% of GastroIntestinal Stromal Tumors, GISTs [3]), promoting the constitutive activation of the KIT receptor without binding to the specific ligand, and leading to uncontrolled proliferation and survival of neoplastic cells [14].

Given the increasing interest in pet dogs as a reliable spontaneous animal model for the study of non-UV-induced melanomas, several authors are also investigating canine melanomas arising in sun-protected sites such as canine oral melanoma (COM), the most frequent neoplasia of the oral cavity in dogs [15,16,17]. Several studies noted some similarities in can profiles between hMMs [10,18,19,20,21] and COMs [10,22,23,24]. In a recent comparative investigation, we were able to detect common chromosomal changes in 32 regions affecting human chromosomes (HSA) and the canine orthologous regions (CFA), with amplification in 35% of cases of the KIT-coding region located on CFA 13 [25].

However, regarding the SNP profile of COMs, the majority of the studies focused almost entirely on Ex-11, trying to compare the results obtained in human medicine without investigating other exons that play a key role in *KIT* activation in hMMs, such as exons 13 (Ex-13), 17 (Ex-17) and 18 (Ex-18) [6,7,8,13,26,27].

When considering the mutations reported in the available human literature, Ex-11 is altered in 67% of hMMs [6,7,8,9,12,13,20,28,29,30,31,32], while Ex-13 is affected in 15.2% [4,6,7,8,9,12,13,20,29,30,31,32], Ex-17 in 12.2% [4,6,7,8,9,13,20,29,30,31,32], and Ex-18 in 8.5% [6,7,13,29,30,32].

In veterinary medicine, only mutations affecting Ex-11 have been adequately investigated in the *KIT* gene, but no decisive results have been achieved, while a screening of Ex-13, Ex-17, and Ex-18 has not been performed [33,34].

The tissue expression of the KIT protein has also been investigated by immunohistochemistry (IHC) in various neoplasms, both in human and veterinary medicine (the latter particularly in pet dogs [35,36,37,38]). Although less documented in COMs, and with different ranges of intensities, 49–51% of neoplasms analyzed expressed the protein [34,39] in contrast to hMMs, where 74–89% of cases were reported to be positive [9,12,26].

These heterogeneous results, both in human and veterinary studies, also bear consequences in the use and effectiveness of therapeutic approaches to *KIT*-bearing mutation tumors.

In humans, the use of tyrosine kinase inhibitors (TKIs) has a better effect on patients affected by tumors bearing SNPs in *KIT* [8,13,27,40], particularly in GIST [41], some types of melanomas [11,20,40], chronic myeloid leukemia [42], and systemic mastocytosis [43].

Moreover, the disease control rate in human patients treated with imatinib, one of the most widely used TKIs, is better in patients bearing tumors with *KIT* point mutations (77%) compared to patients bearing tumors with *KIT* amplification (18%) [40], particularly when SNPs affect Ex-11 and Ex-13 [8,13,27]. For patients with SNPs in Ex-17 and Ex-18, which are not responsive to imatinib, responsiveness to treatment with MEK-1 inhibitors has instead been suggested [8].

TKIs are also successfully used in veterinary medicine for the treatment of Mast Cell Tumors (MCTs) with *KIT* point mutations in Ex-11 [34,44], and occasionally of GIST [45,46]. Moreover, although imatinib appears to be effective mostly for tumors with *KIT* point mutations, disease regression in dogs and cats treated with imatinib and affected by tumors without known *KIT* point mutations is rare [47].

In this study, we investigated the role of *KIT* in COMs at both the gene and protein level. Taking advantage of the cohort of COMs assembled for the array Comparative Genomic Hybridization (aCGH) study published in 2019 [25], we decided to deepen our knowledge of the possible role of the gene *KIT* and the protein KIT in our cohort of samples. We performed an accurate evaluation of the possible SNPs by sequencing Ex-13, Ex-17, and Ex-18, and by evaluating KIT expression in COMs. Ki67 was also evaluated by IHC due to its higher expression in metastatic hMMs with SNPs in *KIT* [13].

Therefore, the aim of this study was to evaluate and correlate the IHC expression of KIT and Ki67 with *KIT* somatic point mutations and amplification in COMs.

## 2. Materials and Methods

### 2.1. First Case Selection

Formalin-fixed, paraffin-embedded (FFPE) COMs were collected from different archives. For each patient, anamnestic data including size and location of the tumor were collected. Hematoxylin and eosin (H&E)-stained slides from each sample were checked by 3 experienced pathologists to confirm the diagnosis of COM and to assess the presence of healthy tissue near the pathological mass suitable for the nucleic acid extraction. The diagnosis of COM was made by adopting the criteria proposed by Smedley and colleagues [48], which were also used for pigmentation scoring, and uncertain cases (i.e., amelanotic specimens) were tested via IHC with anti-PNL-2 and anti-Melan-A antibodies. Only cases that were unanimously diagnosed as COM by all the reviewing pathologists were taken into consideration. At the end of phase one, forty cases met all the inclusion criteria and were selected for the following analyses.

### 2.2. Nucleic Acid Extraction

With the help of a microtome with disposable blades, 2–3 20 µm-thick slides were cut from each FFPE block. Healthy and cancerous tissues were separated using a clean scalpel and put in 2 different tubes. To avoid any possible contamination, all the instruments were cleaned between one sample and the other. From the paraffinized material, DNA was extracted using the All Prep DNA/RNA Extraction Kit (Qiagen, Hilden, Germany^®®^) using heptane as the deparaffinization agent and following the manufacturer’s instructions. The extracted DNA was then evaluated on a 1% agarose gel by electrophoresis and quantified with the NanoDrop ND-1000 (Thermo Fisher Scientific, Waltham, MA, USA^®®^).

### 2.3. aCGH Analysis and Second Case Selection

Twenty of the 40 samples showed an adequate DNA yield and were submitted to an aCGH analysis as described in the work from Brocca and colleagues [25]. The aCGH allowed us to learn the aberrational status of CFA 13, and in particular of the portion containing the *KIT* gene. All the samples affected by a copy number gain in the *KIT* locus were selected for the study, together with an equal number of samples not affected by a copy number gain involving *KIT*. The latter were randomly chosen among the remaining samples submitted to the aCGH analysis to maintain a balanced number of samples between the 2 groups (Table 1).

### 2.4. Exon Amplification and Sequencing

Primers were designed (Table 2) to amplify the exons considered in this study (Ex-13, Ex-17, and Ex-18). For each exon, reference sequences (CanFam3.1 annotation) were obtained from the online platform Ensembl [50], and primers were designed with the software Primer3 v4.1 [51]. Amplicon size and coding sequence covered for each exon are reported in Table 2.

A polymerase chain reaction was then performed for all samples with an initial denaturation step for 2 min at 94 °C, 42 cycles of 40 s at 94 °C (denaturing), 40 s at 60.5 °C (annealing), 50 s at 72 °C (extension), and 5 min of final extension at 72 °C.

The positive control was non-fragmented genomic DNA extracted from fresh-frozen canine cutis, while water was used instead of DNA as the negative control.

All amplicons obtained were separated on a 1.8% agarose gel via electrophoresis to assess the success of the PCR reaction, then purified with ExoSAP (Exonuclease I Shrimp Alkaline Phosphatase, Thermo Fisher Scientific^®®^). Using the corresponding forward (Ex-17) or reverse (Ex-13 and Ex-18) primer, amplicons were then sequenced with the Sanger method, and their sequences were visualized with the Chromas 2.6.5 software. Only sequences with high-resolution peaks (high signal-to-noise ratio), minimal baseline noise, and no trace of secondary sequence contamination were considered suitable for mutational analysis. For each sample, healthy and pathological sequences were matched and aligned with the Clustalw platform [52]. The reference sequence was used to identify SNP positions. The use of pathological and healthy tissue from the same dog allowed for the discrimination of germline and somatic SNPs, and only the latter were taken into consideration for further analyses. The presence of a specific mutation in at least 15% of the samples was arbitrarily considered the minimum threshold.

### 2.5. Immunohistochemistry and Immunohistochemical Assessment

KIT expression was evaluated in each sample included in this study. For each FFPE block, a 4 µm-thick slide was cut, mounted on a polarized glass slide (TOMO^®®^, Matsunami Glass) and then tested with an anti-KIT rabbit polyclonal antibody diluted 1:300 (Dako^®®^, CD117 clone).

For each specimen, the expression of the Ki67 protein was also assessed with an anti-Ki67 mouse monoclonal antibody diluted 1:50 (Dako^®®^, MIB1 clone). Both antibodies were previously validated in the canine species [35,53].

Both procedures were performed with an automatic immunostainer (Ventana Benchmark GX, Roche-Diagnostic). To avoid the use of a bleaching reaction which could damage the integrity of the antigens, an ultraView universal alkaline phosphatase RED detection kit (Ventana Medical System Inc., Oro Valley, AZ, USA) was used (DAB chromogen in unbleached specimens is indeed not usable), and hematoxylin was used as a counterstain.

As positive controls, a canine MCT and canine cutis were used for KIT and Ki67 staining, respectively. As negative controls, antibody diluent was applied instead of the antibody.

**Figure 1:**
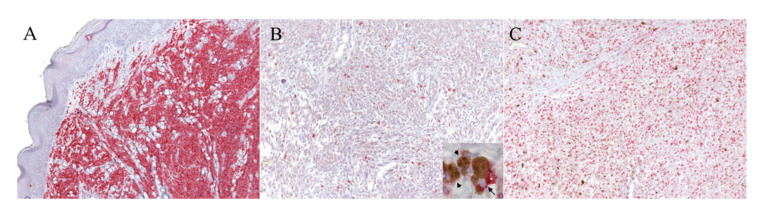
IHC for KIT and Ki67 with the RED labeling system. (**A**) Mast cell tumor (MCT). Example of the mast cell tumor used as the control tissue for the KIT immunolabeling, with a high density of positive cells (100× magnification). (**B**,**C**) Canine oral melanoma (COM). (**B**) Example of a neoplastic area selected for the evaluation of the KIT index, which was 6.6 (100× magnification): the positive cells are discernible and scattered throughout the field. The details of the positive neoplastic melanocytes (arrow) are provided with higher magnification in the inset; melanomacrophages are also present within the inset (arrowheads). (**C**) Example of a neoplastic area selected for the evaluation of the Ki67 index, which was 30 (100× magnification).

The slides were visualized at 40× magnification using a D-Sight scanning machine and the D-Sight Viewer software (A. Menarini Diagnostics). The KIT index was evaluated by counting the mean number of positive cells in 5 consecutive high-power fields (hpf; 0.237 mm^2^) within the areas with clear positive staining, starting from the mostly positive field. If no positive cells were found in 3 consecutive hpf, the process was repeated in another IHC-positive area. This method was designed similarly to that used for the establishment of the Ki67 index as described by Bergin and colleagues [50], which was applied for the Ki67 index calculation. When more than one biopsy was present for each tumor (e.g., for margin evaluation), 5 hpf were selected for each specimen. Only areas with a cellular population representative of the tumor were selected, and areas affected by background, degeneration, scirrhous reaction, or necrosis were avoided. Neoplastic cells were considered positively KIT-labeled when they showed brightly red cytoplasmic and membrane staining, as exemplified in Figure 1A from the MCT control, and as described in the literature [35].

### 2.6. Statistical Analysis

Statistical analyses were performed using MedCalc (MedCalc Statistical Software version 15.8). Data distribution was visually checked for normality. To verify mean differences among groups, either the Student’s *t* test or the one-way ANOVA with Tukey’s multiple comparisons were performed when data were normally distributed. The Mann–Whitney test or Kruskal–Wallis test was applied when data were not normally distributed. A Chi-square test and Fisher’s exact test were used for analysis of the association between *KIT* amplification status and clinical features.

The Spearman’s rank correlation analysis was applied to discover associations between variables. The level of significance was set at *p* < 0.05.

## 3. Results

### 3.1. aCGH Analysis

Data related to the aCGH analysis are published and extensively discussed in the paper from Brocca and colleagues [25]. Twenty samples were submitted for aCGH analysis, and a total number of 14 samples were selected for this study: seven harboring a KIT amplification, and seven without KIT amplification, which were randomly chosen among the remaining 13 samples to maintain a balanced number of samples between the two groups.

### 3.2. Epidemiological Data

The mean age of the 14 dogs selected for the study was 11.98, ranging from 10–17 years. Males represented 78.57% (11/14, one neutered) of the cases, while females represented the remaining 21.43% (3/14, one neutered). Male over-representation is in accordance with the literature [15,54]. The most represented breed was the cocker spaniel, with 28.57% (4/14), and the most common sites of the neoplasia (when specified) was the oral lip, with 57.14% (8/14, six of which on the upper lip). For the immunohistochemical evaluation of Ki67, the majority of the samples (85.71%, 12/14) showed an index >19.5, significant of a bad prognosis at one-year post-diagnosis [50]. This data is visible in Table 1. All samples came from surgical excision or incisional biopsy.

### 3.3. Identification of Somatic Mutations

**Figure 2:**
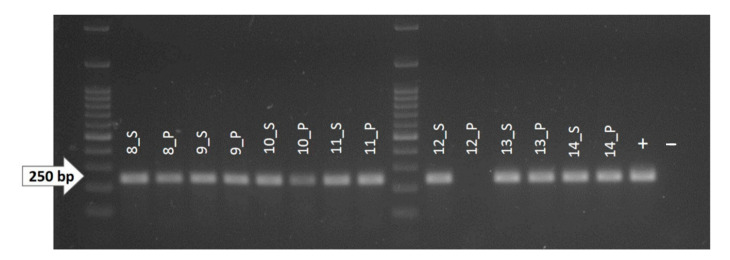
1.8% agarose gel showing the amplicons obtained by PCR reaction for exon 13 (Ex-13) of the KIT gene. As shown, no amplified DNA was obtained from the pathological fraction of sample 12 (12_P). +: positive control (non-fragmented canine genomic DNA); −: negative control (water).

The DNA from all 14 samples was successfully extracted from the fractions of healthy and pathological tissues, with the use of heptane as the deparaffinizing agent to obtain a higher yield of extracted nucleic acids. From Nanodrop analysis, healthy tissues showed a lower amount of extracted DNA (*p* < 0.0001, data not shown), and the 260/280 and 260/230 ratios showed variable values, but all were considered of sufficient quality for the amplification steps. All primer pairs were firstly tested on canine control tissues at different temperatures with a gradient PCR, and all pairs showed successful and specific amplification of the selected exons. The amplification was successful in almost all samples for both the healthy and the pathologic tissues. A band at the expected length was obtained in 27/28 (96.4%) reactions (14 DNA samples from healthy tissues and 14 DNA samples from pathologic tissues) for exon 13, 27/28 (96.4%) for exon 17, and 28/28 (100%) for exon 18 (Table 3). Indeed, it was not possible to obtain an amplification product for exons 13 or 17 using the pathologic DNA extracted from sample 12 (Figure 2). All amplicons obtained were successfully sequenced and analyzed as described by three operators. In summary, 97.6% of the exonic sequences (82 sequences out of 84) examined were successfully amplified and Sanger-sequenced, and no somatic SNPs, insertions, or deletions were identified in any of the samples.

### 3.4. Immunohistochemistry

One of the samples (sample 7 in Table 1) could not be evaluated for KIT expression since the DNA extraction procedure exhausted the paraffin block.

Positive immunolabeling was obtained in the MCT control (Figure 1A) and in all the cutaneous melanocytes (internal control) of the healthy tissues included along with COM specimens.

As expected, the anti-KIT antibody showed cytoplasmic and membranous labeling of neoplastic melanocytes with a bright multifocal positivity of a scarce to a moderate number of cells (Figure 1B). The positive cells were discernible and randomly distributed across the tumor tissue in the majority of samples; particular patterns of distribution were not noted. Only two samples (15.4%) did not show any staining for KIT expression.

The KIT index varied from 0.6 to 8.6 in positive samples, with a mean of 3.1 positive cells in 5 hpf. Positive staining for the anti-Ki67 antibody (Figure 1C) was obtained for all specimens and the Ki67 index was established as described [50]. The Ki67 index varied from 7.6 to 265, with a mean of 62.4 positive cells in 5 hpf. According to the Ki67 prognostic cut-off of 19.5, three samples were classified as having a good prognosis (G), and the remaining 11 as having a bad (B) prognosis. The results are summarized in Table 1. When samples were divided into two groups according to the KIT amplification status, no statistically significant differences were detected either for the KIT index (*p* = 0.56) or for the Ki67 index (*p* = 0.38). Similarly, no differences in terms of KIT and Ki67 expression were noted between males and females (*p* = 0.87 and *p* = 0.46, respectively), or according to the pigmentation level (*p* = 0.48 and *p* = 0.73, respectively). Finally, no correlation was found between the two indexes (*r* = 0.074, *p* = 0.81).

## 4. Discussion

The aim of this study was to deepen our understanding of the mutational landscape of the *KIT* gene in COMs, particularly in exons 13, 17, and 18, and to correlate the mutational profile of these exons with the amplification status of the gene itself, and with the IHC expression of the KIT protein.

This interest derives from the scarcity of currently available similar studies in the canine species, and from the possible use of pet dogs as a reliable model for the study of hMMs.

In our previous work [25] that aimed to improve our knowledge about the genomic DNA alterations that occur in COM, many genes related to MAPK and PI3K pathways were detected from the CNA analysis, together with a wide variety of genes coding for tyrosine kinases receptors, including *KIT*. Interestingly, the pathway enrichment analysis revealed the enhancement of pathways specifically related to cancer proliferation, but also a significant enrichment of those related to imatinib and drug metabolism. These results indicated that further investigation of the *KIT* alteration status was warranted.

In this work, we describe the first characterization of the mutational profile of exons 13, 17, and 18 of the *KIT* gene in COM, which were successfully PCR amplified and Sanger-sequenced. Taking advantage of the cohort of samples collected for the aCGH study [25], we were able to compare the exon sequences of healthy and pathologic tissues from COMs with known *KIT* amplification status. In particular, CFA 13 (comprising the *KIT* gene) was affected by a copy number gain in 7/20 samples of the original cohort (35%), and we considered it valuable to further analyze the DNA of these seven samples and to compare them with another randomly chosen seven samples that were not affected by the same copy number gain in CFA 13.

We developed highly-performing primer pairs and set up a reliable protocol for the amplification and sequencing of short genomic sequences extracted from FFPE blocks.

Since no SNPs were detected affecting the examined exons, our study suggests that *KIT* status in COMs does not resemble the mutational status reported in hMMs. This is in line with some of the latest Next Generation Sequencing-based veterinary studies [10,24]. Although Garrido and Bastian [4] suggested that CNAs and SNPs in hMM are mostly mutually exclusive, two different studies reported *KIT* amplification and coexisting SNPs in exons 11, 13, 17 and 18 in the same tumor [6,8].

In our study, none of the samples affected by a CFA 13 amplification had an SNP present, and no point mutations have been found at all, suggesting one possible molecular difference between COMs and hMMs.

The absence of point mutations in our cohort is consistent with the results reported in other recent studies of COMs, in which the *KIT* point mutations are considered a sporadic event [10,33,34,47], which highlights a potential significant molecular difference with hMMs [4,8,10].

The detection of a 35% prevalence of *KIT* amplification versus a 0% prevalence of *KIT* point mutations in our cohort of COMs corroborates the increasingly affirmed hypothesis that the main pathogenesis of COMs and hMMs is related predominantly to CNAs rather than SNPs [10,18,19,20,21].

Regarding the pathologic DNA from sample 12, we were not able to obtain an amplification reaction for exon 13 and 17, while the amplification of exon 18 was successful (Table 3). The reason for this could be the high melanin content of the sample. Indeed, melanin is an interferer of the PCR reaction and other molecular analysis when it is co-purified in the process of DNA extraction. Moreover, it has already been demonstrated that PCRs producing longer amplicons are more inclined to be inhibited by melanin than PCRs producing amplicons of shorter size [55]. In our case, melanin could have bound to the DNA polymerase enzyme, preventing the PCR reaction in the longer exons, i.e., exons 13 and 17, which both had a length close to 250 bp, but not in the shorter exon 18, which is approximately 200 bp long.

Here, we proposed a reproducible method for scoring KIT IHC positivity in COMs samples. To date, the IHC evaluation of KIT expression has been limited to a semi-quantitative evaluation, expressed as classes corresponding to an approximate percentage of immunoreactive cells on the total tumor area, often with wide ranges defining a single class [34,39]. In our opinion, this approach poorly describes the mutable status of expression of the protein. In support of this hypothesis and in contrast with other studies, our IHC results closely reflect the human literature: 84.6% of our samples were indeed positive, and therefore nearer to the percentage provided in human literature. In other veterinary studies, only about half of the COMs examined (49–51%) [34,39] were considered positive with the semi-quantitative scoring method. It is still unclear if this difference is related to the different scoring method or to a real difference in KIT expression between hMMs and COMs, and further evaluation (or a re-evaluation of previous works) is necessary. We also semi-quantitatively scored our samples following the methods proposed in [34,39], and we noted an overestimation of negative cases when the percentage of positive cells was <10% (data not shown).

There was no significant difference in KIT protein expression between *KIT* amplified and non-amplified samples. This could be due to the low number of cases analyzed, or to the fact that *KIT* gene amplification does not correspond to a higher KIT protein expression.

As reported by Lassam and Bickford [56], and by Montone and colleagues [57], an interesting observation regarding KIT in melanomas is the decrease (or even the loss) of KIT expression along with the progression of the neoplastic disease. This was observed in human cultured melanoma cells [56] and in cutaneous melanomas (from radial growth phase to vertical growth phase and metastatic melanoma) [57], which led to the hypothesis that the loss of KIT could represent a negative prognostic factor [56,57].

These findings support the hypothesis of Alexeev and Yoon [5], who proposed that for malignant melanocytes to acquire metastatic potential and escape from the epidermal boundaries [4], they necessarily have to lose KIT expression.

Indeed, a study from Newman et al. [39] found a significant association between the presence of KIT IHC positivity and patient survival, suggesting that the downregulation or loss of KIT could be related to increasing invasiveness in dogs as well.

Regarding the correlation with other tumor markers, Ma and colleagues [13] highlighted an increased Ki67 expression in metastatic hMM, while the lack of pigment was considered a negative prognostic factor by Prouteau and colleagues [58]. However, in our study, no statistically significant difference was noted in either case.

Unfortunately, follow-up data were not available for this cohort of samples, making further evaluations impossible.

## 5. Conclusions

In this paper, we showed that exons 13, 17, and 18 of the *KIT* gene do not present with SNPs in our cohort of COMs, suggesting that they are not involved in the pathogenesis or progression of COM. Moreover, the amplified status was not statistically associated in any way to a mutational profile in the *KIT* gene, nor to the expression of the KIT protein itself or to the Ki67 marker.

Immunohistochemically, we proposed a quantitative, replicable method for the evaluation of KIT expression, through which 84.6% of the samples (11/13) were detected to be positive for KIT. Although the number of cases included in this study did not allow us to draw definitive conclusions, our findings provide new insights for the current knowledge on the use of pet dogs as a spontaneous model for the study of hMMs. Further studies with a greater number of cases are needed to clarify unresolved questions, including the role played by other kinases that have also been found to be altered in our cohort via aCGH analysis, such as *PTK2*, *STK3*, *TEC*, *PDGFRA*, *VEGFR2*, and *CD63*, which so far have received less attention.

## Figures and Tables

**Table 1:** Selected samples included in the study, together with available clinical and histological data, immunohistochemistry (IHC) indexes, and amplification status. B: bad prognosis; F: female; FN: neutered female; G: good prognosis; M: male; MN: neutered male; NA: not available; * mean number of positive cells in 5 high-power fields (hpf).

Case ID	Site (Oral Cavity)	Breed	Age (Years)	Sex	Pigmentation [48]	*KIT* locus Amplification	Prognosis [49]	Ki67 Index * [49]	KIT Index *
1	Mandible	West Highland White Terrier	11	M	<50%	yes	G	16.2	8.6
2	Cheek	Cross breed	17	M	<50%	yes	B	137.4	0.8
3	Oral	Rottweiler	12	FN	<50%	no	B	29.6	1.4
4	Lip	American Cocker Spaniel	10	MN	<50%	yes	B	54.2	5
5	Upper lip	Golden Retriever	12	M	<50%	yes	B	113.4	6.6
6	Lip	Cocker Spaniel	10	F	<50%	no	B	26.2	6.6
7	Mandible	Pug	11	M	<50%	yes	B	37.4	NA
8	Oral	Collie	11	M	≥50%	yes	B	30	0
9	Upper lip	German Shepherd	11	M	<50%	yes	B	22.6	0.6
10	Mandible	Cocker Spaniel	11	F	≥50%	no	B	21.6	2
11	Upper lip	Pinscher	17	M	≥50%	no	G	7.6	3
12	Upper lip	Cocker Spaniel	10	M	≥50%	no	B	265	3.8
13	Upper lip	Basset Hound	11	M	<50%	no	G	9.8	0
14	Upper lip	Golden Retriever	13	M	<50%	no	B	102.4	1.6
							**Mean**	62.4	3.1

**Table 2:** Primers designed for the amplification of exons 13, 17, and 18 of the *KIT* gene with the relative lengths. CDS: Coding DNA Sequence; F: forward; R: reverse.

		Primer Sequence	Primer Length	Annealing Temp	Primer Genomic Location	Amplicon Size (bp)	CDS Covered (%)
**Exon 13**	F Primer	TGGCTTGCCAAATTTGCTTCT	21	59.58° C	13:47108547–47108567	247 bp	100%
	R Primer	AACCAAGCACTGTCGCAATG	20	59.69 °C	13:47108774–47108793		
**Exon 17**	F Primer	TGACATAGCAGCATTCTCGTGT	22	60.09 °C	13:47113635–47113656	257 bp	100%
	R Primer	TCCTTCACTGGACTGTCAAGC	21	59.93 °C	13:47113871–47113891		
**Exon 18**	R Primer	CATTGCCGGATCTGTTGTGC	20	60.18 °C	13:47117315–47117334	211 bp	100%
	F Primer	AGGACCCTGCTAACCCCTTA	20	59.58 °C	13:47117506–47117525		

**Table 3:** Result of the PCR amplification obtained for each DNA fraction (healthy and pathologic) of the samples analyzed, and for each primer pair. H: healthy; P: pathologic. Ex: exon.

Case ID	DNA	Ex-13	Ex-17	Ex-18
1	H	yes	yes	yes
	P	yes	yes	yes
2	H	yes	yes	yes
	P	yes	yes	yes
3	H	yes	yes	yes
	P	yes	yes	yes
4	H	yes	yes	yes
	P	yes	yes	yes
5	H	yes	yes	yes
	P	yes	yes	yes
6	H	yes	yes	yes
	P	yes	yes	yes
7	H	yes	yes	yes
	P	yes	yes	yes
8	H	yes	yes	yes
	P	yes	yes	yes
9	H	yes	yes	yes
	P	yes	yes	yes
10	H	yes	yes	yes
	P	yes	yes	yes
11	H	yes	yes	yes
	P	yes	yes	yes
12	H	yes	yes	yes
	P	no	no	yes
13	H	yes	yes	yes
	P	yes	yes	yes
14	H	yes	yes	yes
	P	yes	yes	yes
